# The Exon Junction Complex Controls the Efficient and Faithful Splicing of a Subset of Transcripts Involved in Mitotic Cell-Cycle Progression

**DOI:** 10.3390/ijms17081153

**Published:** 2016-08-02

**Authors:** Kazuhiro Fukumura, Shunichi Wakabayashi, Naoyuki Kataoka, Hiroshi Sakamoto, Yutaka Suzuki, Kenta Nakai, Akila Mayeda, Kunio Inoue

**Affiliations:** 1 Department of Biology, Graduate School of Science, Kobe University, 1-1 Rokkodaicho, Nadaku, Kobe 657-8501, Japan; hsaka@kobe-u.ac.jp (H.S.); kunio@kobe-u.ac.jp (K.I.); 2 Division of Gene Expression Mechanism, Institute for Comprehensive Medical Science (ICMS), Fujita Health University, Toyoake, Aichi 470-1192, Japan; mayeda@fujita-hu.ac.jp; 3 Human Genome Center, Institute of Medical Science, The University of Tokyo, 4-6-1 Shirokanedai, Minato-ku, Tokyo 108-8639, Japan; s-wakaba@hgc.jp (S.W.); knakai@ims.u-tokyo.ac.jp (K.N.); 4 Department of Computational Biology and Medical Sciences, Graduate School of Frontier Sciences, The University of Tokyo, Kashiwa, Chiba 277-8561, Japan; ysuzuki@k.u-tokyo.ac.jp; 5 Laboratory for Malignancy Control Research, Medical Innovation Center, Kyoto University Graduate School of Medicine, Kyoto 606-8507, Japan; 6 Laboratory of Cell Regulation, Departments of Applied Animal Sciences and Applied Biological Chemistry, Graduate School of Agriculture and Life Sciences, The University of Tokyo, 1-1-1 Yayoi, Bunkyo-ku, Tokyo 113-8657, Japan; kataoka.naoyuki.6m@kyoto-u.ac.jp

**Keywords:** exon junction complex (EJC), Y14, pre-mRNA splicing, mitotic cell-cycle

## Abstract

The exon junction complex (EJC) that is deposited onto spliced mRNAs upstream of exon–exon junctions plays important roles in multiple post-splicing gene expression events, such as mRNA export, surveillance, localization, and translation. However, a direct role for the human EJC in pre-mRNA splicing has not been fully understood. Using HeLa cells, we depleted one of the EJC core components, Y14, and the resulting transcriptome was analyzed by deep sequencing (RNA-Seq) and confirmed by RT–PCR. We found that Y14 is required for efficient and faithful splicing of a group of transcripts that is enriched in short intron-containing genes involved in mitotic cell-cycle progression. Tethering of EJC core components (Y14, eIF4AIII or MAGOH) to a model reporter pre-mRNA harboring a short intron showed that these core components are prerequisites for the splicing activation. Taken together, we conclude that the EJC core assembled on pre-mRNA is critical for efficient and faithful splicing of a specific subset of short introns in mitotic cell cycle-related genes.

## 1. Introduction

Pre-mRNA splicing, the correct and precise removal of introns is an essential part of gene expression in eukaryotes. The spliceosome, which catalyzes pre-mRNA splicing, deposits a multi-protein complex, called the exon junction complex (EJC), onto spliced mRNAs ~24 nucleotides (nt) upstream of exon–exon junctions in a sequence-independent manner (reviewed in [1]). The EJC is composed of four core components, eIF4AIII, Y14, MAGOH and MLN51, and many proteins that are weakly associated with the EJC core, termed EJC peripheral factors. In metazoans, the EJC core functions as a binding platform for more than a dozen peripheral protein factors that allow it to regulate multiple subsequent post-splicing gene expression events including mRNA export, mRNA localization, translation, and mRNA surveillance via nonsense-mediated mRNA decay (NMD). The well-characterized representatives of EJC peripheral factors are Aly/REF, UAP56, NXF1/TAP and NXT1/p15 that are involved in mRNA export, and UPF1, UPF2, UPF3 and SMG6 that are essential factors for NMD.

Notably, the EJC peripheral factors also include several splicing regulatory proteins such as RNPS1, ACINUS, SAP18 and PININ (reviewed in [1]). RNPS1 was originally identified as a general splicing activator in vitro and as a regulator of alternative splicing in vivo [2,3,4]. RNPS1 also has roles in the 3′-end processing, translation and NMD [5,6,7]. It is known that ACINUS is involved in apoptosis, RNA processing and transcriptional regulation [8,9]. SAP18 was identified as a component of the Sin3 histone deacetylase complex that enhances transcriptional repression [10], but SAP18 is also capable of modulating alternative splicing via its ubiquitin-like fold [11]. PININ was originally identified as a desmosome-associated protein [12], but it also functions as a splicing co-activator [13]. RNPS1, SAP18, and ACINUS were identified as a ternary complex termed the apoptosis and splicing-associated protein (ASAP) complex [8]. Moreover, a recent structural analysis showed that RNPS1 and SAP18 interact with PININ, forming another ternary complex, PSAP [14]. However, it still remains unclear whether these ternary complexes are associated with the EJC core. Interestingly, it was shown that core and peripheral EJC components regulate alternative splicing of *BCL-X* pre-mRNA through its binding to a *cis*-acting element, whose activity is distinct from the established EJC function [15]. All this evidence suggested that the EJC core is capable of recruiting various splicing regulators and that these interactions may indeed regulate pre-mRNA splicing.

In this study, we performed an siRNA-mediated depletion of an EJC core factor, Y14, followed by whole transcriptome analysis to identify the introns affected by Y14. Intriguingly, we found that Y14 plays a critical role in the efficient and faithful splicing of a particular group of transcripts including short introns, many of which are involved in mitotic cell-cycle progression. Accordingly, knockdown of Y14 induced G2/M arrest and apoptosis in HeLa cells. Furthermore, a tethering assay of the EJC core components (eIF4AIII, Y14 or MAGOH) demonstrated that the formation of the EJC core onto pre-mRNA (not onto mRNA) enhances splicing. These results provide a considerable insight into the EJC-mediated splicing fine-tuning mechanism for short introns in functionally related genes.

## 2. Results

### 2.1. The EJC Core Component Y14 Is Required for Efficient and Faithful Splicing of a Subset of Transcripts

To investigate whether the EJC is implicated in pre-mRNA splicing, we performed a deep-sequencing analysis of transcriptome in Y14-knockdown HeLa cells, i.e., RNA-Seq analysis. DNA libraries were prepared with poly(A)+ mRNA isolated from total RNA (DNase-digested), which is derived from HeLa cells treated with Y14 siRNA or control siRNA. The resulting reads were aligned to the human genome reference sequence using the TopHat mapping tool. To examine the splicing efficiency, we calculated the intron retention rate (IRR) from the RNA-Seq data sets from control siRNA- and Y14 siRNA-treated HeLa cells as described in Supplementary Experimental Procedures.

**Figure 1:**
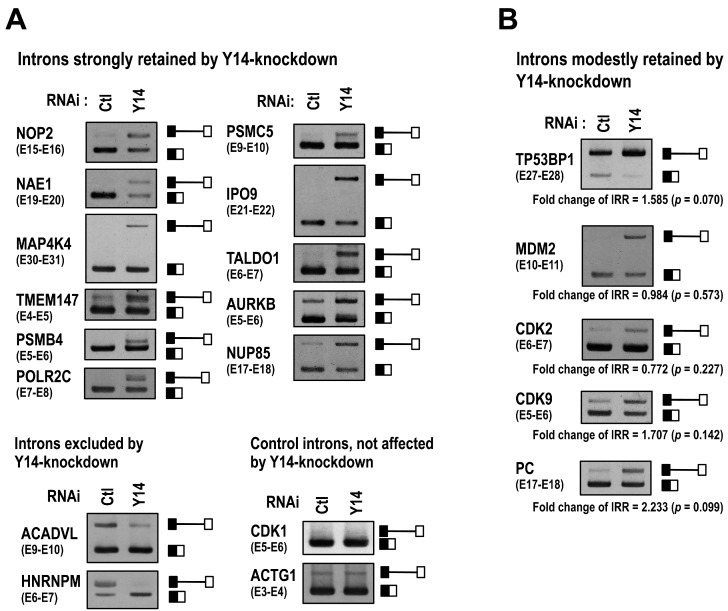
A subset of introns are retained upon Y14 knockdown. RNA-Seq-based selection was performed with total RNAs that were prepared from control siRNA- or Y14 siRNA-treated HeLa cells. The selected representative introns were analyzed by RT–PCR (see Supplementary Table S4 for primer sequences). All PCR products were subcloned and the sequences were verified. The schematic representation on the right of each panel indicates the corresponding unspliced- and spliced-products. (**A**) The upper and lower left panels represent the IRR high score group (log _2_ [Y14 IRR/Ctl IRR] ≥ 1.0, *p* < 0.05) and low score group (log _2_ [Y14 IRR/Ctl IRR] ≤ −1.0, *p* < 0.05), respectively. The lower right panel shows the control group with *CDK1* (E5–E6) and *ACTG1* (E3–E4) pre-mRNAs; their splicing efficiencies were not changed either in Y14 siRNA- or in control siRNA-treated cells; and (**B**) these five selected introns were categorized in the following IRR score group (0 < log _2_ [Y14 IRR/Ctl IRR] < 1, *p* < 0.05 or log _2_ [Y14 IRR/Ctl IRR] ≥ 1.0, *p* > 0.05), in which RT–PCR analyses showed apparent intron retention in sY14 siRNA-treated cells.

As a result, we found that 626 introns in 483 genes were retained at higher levels (IRR high score group) in Y14-knockdown HeLa cells (Supplementary Table S1A,B). In contrast, 335 introns in 250 genes were retained at lower levels (IRR low score group) in Y14-knockdown HeLa cells (Supplementary Table S1C,D). We selected 15 introns, and the RNA-Seq data were validated by RT–PCR (Figure 1A; 11 introns from the IRR high score group, two introns from the IRR low score group, and two introns as controls). We also identified five introns of which splicing was moderately inhibited in Y14-knockdown HeLa cells according to our RNA-Seq data sets (Figure 1B). These results indicated that the splicing efficiency of some specific introns is enhanced by Y14, but splicing of other specific introns is repressed. Moreover, we found that knockdown of another EJC core component, eIF4AIII, has similar splicing repressive effect on Y14-knockdown responsive introns (Supplementary Figure S1). Taken together, we conclude that the EJC core selectively affects the splicing efficiency of some particular, but not all, introns.

**Figure 2:**
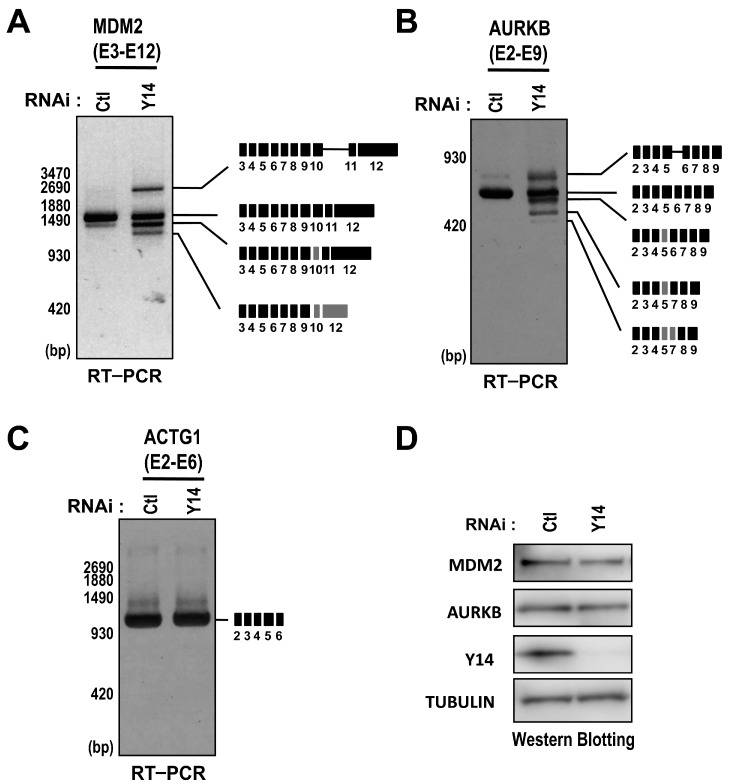
Y14 is required for faithful splicing of MDM2 and AURKB pre-mRNAs. (**A**–**C**) HeLa cells were transfected with control siRNA or Y14 siRNA and obtained total RNAs at 48 h post-transfection were analyzed by RT–PCR using primer sets for MDM2 (**A**), AURKB (**B**), and ACTG1 (**C**) transcripts. The RT–PCR products were subcloned and the sequences were verified. The schematic representation on the right indicates the corresponding mRNA products. Black boxes represent full-length exons and grey boxes represent truncated exons generated by alternative splice site usage; and (**D**) Western blot analysis of whole cell extracts of control siRNAs- or Y14 siRNA-treated HeLa cell using anti-Y14, anti-MDM2, anti-AURKB, and anti-TUBULIN antibodies.

To examine the role of the EJC in efficient pre-mRNA splicing, we focused on a set of retained introns in Y14-knockdown HeLa cells. At first, we investigated the transcripts from the *AURKB* (Aurora B kinase), *MDM2* (murine double minute2) and *ACTG1* (actin γ1) genes in Y14-knockdown cells. We tested whether the intron retention would be accompanied by the aberrant splicing, generating the abnormal mRNAs. Interestingly, Y14 knockdown resulted in the reduction of intact mRNAs accompanied by the production of several abnormal mRNAs from the *MDM2* and *AURKB* genes (Figure 2A,B), while only the full-length transcript from the *ACTG1* gene was detected in Y14-knockdown HeLa cells (Figure 2C). Sequencing of truncated transcripts for the *MDM2* and *AURKB* genes confirmed that aberrant splicing and exon skipping occurred in Y14-knockdown HeLa cells (Figure 2A,B). These abnormal transcripts might be translated into the proteins that could be deleterious for cells, although we found the amounts of MDM2 and AURKB proteins were largely unaffected (Figure 2D). These results suggested that the EJC contributes to the efficient and proper pre-mRNA splicing of a subset of transcripts.

### 2.2. The Targets of Y14-Mediated Splicing Activation Are Short Introns in Genes Involved in Cell Cycle Progression

**Figure 3:**
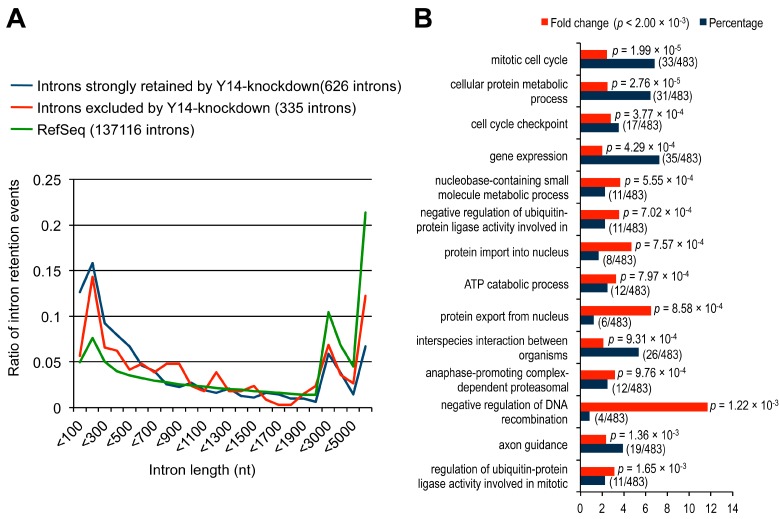
A majority of retained introns in Y14 knockdown cells are short (<500 nt) and exist in a subset of genes involved in cell cycle and apoptosis. (**A**) Retained introns in control siRNA-, Y14 siRNA-treated HeLa cells and RefSeq were grouped by length and plotted as a ratio to the total introns; and (**B**) gene ontology (GO) analysis indicated the functions of 483 genes that showed intron retention inY14 siRNA-treated HeLa cells.

It has been reported that the EJC components play an important role in proper splicing of transcripts containing long introns (>1000 nt) in *Drosophila* [16,17]. To examine if this is the case in mammalian cells, we investigated the size distribution of the Y14-knockdown responsive introns. Remarkably, 52.4% (328/626) of the introns in the IRR high score group, in which splicing was strongly inhibited in Y14-knockdown cells, were shorter than 500 nt (Figure 3A and Supplementary Table S2). The ratios of the shorter introns (<500 nt) in the IRR low score group and a control Ref-seq group were 37.0% (124/335) and 25.1% (34422/137116), respectively, which are significantly lower than the ratio in the IRR high score group. These results suggest that the EJC has a critical role in efficient splicing of pre-mRNAs with short introns in mammals, in stark contrast to the EJC-sensitive splicing defect of long introns in *Drosophila*.

Next, we investigated the biological function of the 483 genes, containing 626 introns in the IRR high score group. We found that the enriched functional categories are related to mitotic cell cycle progression, including mitotic cell cycle (33/483), cell cycle check point (17/483), negative regulation of ubiquitin-protein ligase activity in mitotic cell cycle (11/483), anaphase-promoting complex-dependent proteasomal ubiquitin-dependent protein catabolic process (12/483), and regulation of ubiquitin-protein ligase activity involved in mitotic cell cycle (11/483) (Figure 3B and Supplementary Table S3). Therefore, we assumed that intron retention might disrupt proper cell cycle progression in Y14-knockdown HeLa cells. Indeed, previous studies had shown that depletion of several EJC components induces the abnormal mitotic spindle formation, genome instability, and apoptosis [15,18]. Consistent with these observations, we found that Y14-knockdown caused abnormal nuclear structures and multinuclear phenotypes, which reflected a disruption of normal cell cycle progression (Supplementary Figure S2A). To examine cell cycle progression, we employed a FACS (fluorescence-activated cell sorting) analysis and found an increase in the population of cells at the G2/M and sub G0/G1 phases in Y14-knockdown HeLa cells (Supplementary Figure S2B). Furthermore, we investigated genome stability by staining Y14-knockdown cells with an antibody against Ser139-phosphorylated histone H2A.X (γH2A.X), the marker for double-strand DNA breaks, and we found that Y14-knockdown induced an increase in H2A.X foci (Supplementary Figure S2C). Taken together, we conclude that the EJC plays a crucial role for efficient and faithful pre-mRNA splicing of the genes that are involved in mitosis and genome stability.

### 2.3. The Binding of the EJC Core Is Required for Splicing Activation of a Model Pre-mRNA

**Figure 4:**
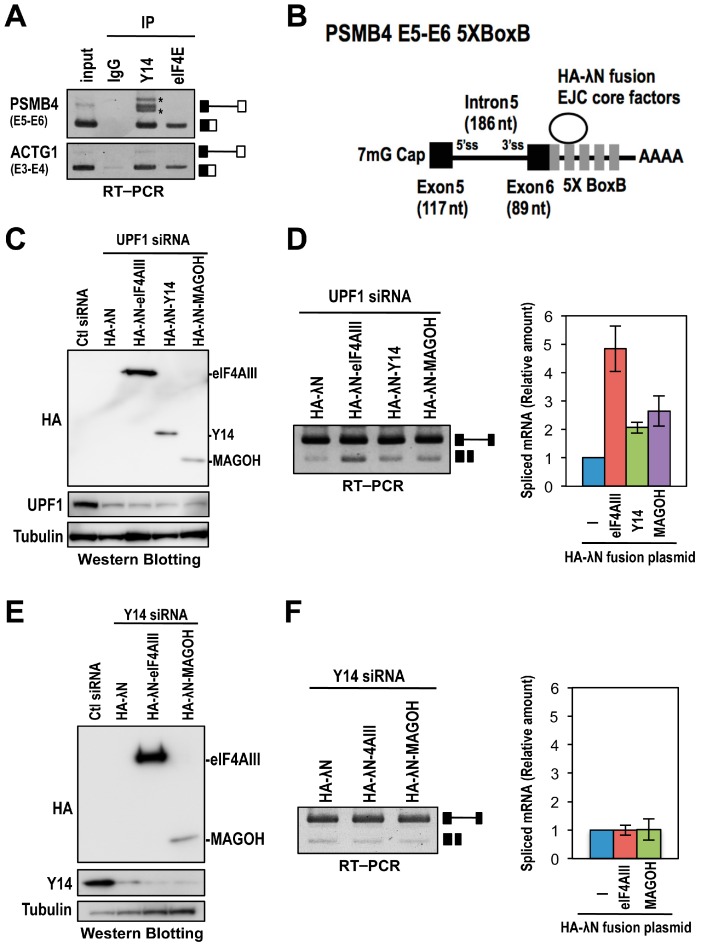
Core EJC assembly is required for increased splicing efficiency of the mini-*PSMB4* model pre-mRNA. (**A**) Whole HeLa cell extracts were subjected to immunoprecipitation (IP) using anti-Y14 or anti-eIF4E antibody in the absence of RNase A. Total RNAs (5% of input) and co-precipitated RNAs were analyzed by RT–PCR using primer sets for *PSMB4* (E5–E6) and *ACTG1* (E3–E4). The schematic representation on the right indicates the corresponding unspliced- and spliced-products. Asterisk (*) indicates non-specific PCR products; (**B**) The schematic representation of a model *PSMB4* exon 5–exon 6 pre-mRNA fused with five BoxB sites in the downstream of exon 6. The HA-λN tagged EJC core components (eIF4AIII, Y14 and MAGOH) are represented by the oval; (**C**) Western blot analysis of expressed HA-λN fusion proteins and endogenous UPF1 protein. UPF1 siRNA-treated HeLa cells were transfected with the *PSMB4* E5–E6-5×BoxB mini-gene plasmids and the indicated HA-λN-tagged EJC component plasmids; (**D**) RT–PCR analysis was performed to detect the unspliced- and spliced- products from the *PSMB4* E5–E6 mini-gene. All experiments were independently repeated three times. Averages and standard deviations of the relative amount of spliced mRNA are shown in the right panel; and (**E**,**F**) the same tethering experiments as (**C**,**D**) using Y14 siRNA-treated HeLa cells.

The EJC is usually forms onto spliced mRNA, however, our implication of the EJC in splicing postulates its association with pre-mRNA. To examine whether the assembly of the EJC core onto pre-mRNA is required for the efficient splicing of Y14-knockdown responsive introns, we used the *PSMB4* (proteasome subunit, β type 4) gene as a model [19]. The *PSMB4* intron 5 is a typical short intron (186 nt), which is retained in Y14- and eIF4AIII-knockdown HeLa cells (Figure 1A and Supplementary Figure S1). We first confirmed the Y14 association with *PSMB4* pre-mRNA containing intron 5 by immunoprecipitation using the Y14 antibody. As expected, Y14 strongly associated with the intron 5-harboring pre-mRNA as well as the intron 5-excised mRNA. On the other hand, the translation initiation factor eIF4E only associated with the spliced mRNA (Figure 4A). These results suggested that the EJC is indeed formed on *PSMB4* pre-mRNA.

We next investigated whether the EJC could increase the splicing efficiency of *PSMB4* pre-mRNA with intron 5. We employed a tethering assay using the λN-BoxB system, which uses the λN peptide to tether the protein of interest to RNAs [20]. We constructed the *PSMB4* exon 5–exon 6 mini-gene fused with five copies of BoxB sequences at the 3′ terminus of exon 6 and the effecter plasmids encoding HA-λN tagged EJC core components (eIF4AIII, Y14 or MAGOH) (Figure 4B). To prevent the NMD-degradation of RNA products from the *PSMB4* mini-gene during this tethering assay, we performed the experiment in the context of siRNA-mediated UPF1 knockdown that represses NMD [20]. Western blotting was performed to check the protein expression levels of HA-λN tagged EJC components as well as the depletion efficiency of endogenous UPF1. The experiments showed that protein expression level of HA-λN-eIF4AIII was higher than those of HA-λN-Y14 and HA-λN-MAGOH under the efficient depletion of endogenous UPF1 (Figure 4C). We then performed RT–PCR to examine the splicing efficiency of the *PSMB4* reporter transcript. Splicing efficiency was increased when the pre-mRNA was tethered with the EJC core components (Figure 4D). We observed that splicing activation by eIF4AIII (approximately five-fold increase compared to HA-λN control) was higher than that caused by Y14 or MAGOH (approximately two-fold increase compared to HA-λN control). It is thus likely that this difference of splicing activation was due to the expression levels of the tethered proteins. In these tethering experiments, we assume that one of the tethered EJC core factors (eIF4AIII, Y14, or MAGOH) would be able to associate with the rest of the endogenous EJC core factors. Next, we performed the tethering of eIF4AIII or MAGOH to *PSMB4* reporter transcripts in Y14-knockdown HeLa cells, where it was expected that neither tethered eIF4AIII nor MAGOH could form the EJC core. Western blot analysis confirmed the protein expression levels of HA-λN-eIF4AIII and HA-λN-MAGOH, and the depletion of endogenous Y14 (Figure 4E). RT–PCR analysis revealed that splicing efficiencies of *PSMB4* reporter transcripts tethered with eIF4AIII or MAGOH were no longer enhanced in the absence of Y14 (Figure 4F). These results suggest that the EJC core formation on pre-mRNA is a prerequisite for observed splicing activation.

### 2.4. RNPS1 Is a Key Factor in EJC Core-Mediated Splicing Activation

**Figure 5:**
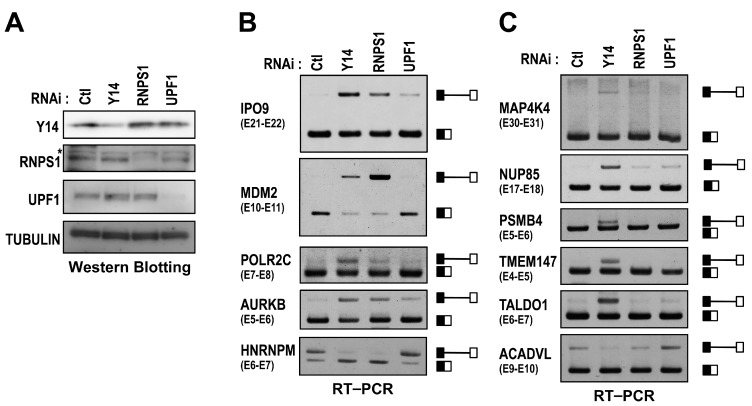
RNPS1 is required for efficient removal of some, but not all, EJC core-responsive introns. (**A**) HeLa cells were transfected with specific siRNAs against Y14, RNPS1, UPF1, or control (Ctl). At 48 h post-transfection, whole cell extracts were subjected to Western blot analysis. The asterisk (*) indicates a non-specific signal; and (**B**,**C**) total RNAs were isolated and analyzed by RT–PCR using primer sets for MDM2, AURKB and ACTG1 (shown in Supplementary Table S4). The schematic representation on the right of each panel indicates the corresponding unspliced- and spliced-products.

Our results indicated that the EJC core recruits a *trans*-acting factor to activate splicing. The EJC core has been reported to associate with accessory factors involved in pre-mRNA splicing, mRNA export, NMD, and translation (reviewed in [1]). To identify the EJC-recruited accessory proteins that promote the efficient splicing, we performed siRNA-mediated knockdown of RNPS1 (splicing activator/regulator) and UPF1 (essential NMD factor). Western blot analysis confirmed that Y14, RNPS1 and UPF1 were efficiently depleted (Figure 5A). Interestingly, RT–PCR analysis showed that RNPS1 knockdown induced the retention of several (Figure 5B), but not all (Figure 5C), Y14-knockdown responsive introns (Figure 5B). In contrast, UPF1 knockdown did not induce intron retention, indicating that intron retentions by Y14 or RNPS1 knockdown are not due to the survival of transcript generated by the loss of NMD function. In addition, we checked the transcripts of *MDM2*, *AURKB* and *ACTG1* by RT–PCR in RNPS1- or UPF1-knockdown HeLa cells. Here, we observed abnormal transcripts of *MDM2* and *AURKB* (both contain Y14-knockdown responsive introns) and concomitant reduction of AURKB protein level in RNPS1-knockdown cells (Supplementary Figure S3A,B,D). In contrast, we detected only the full-length transcript of *ACTG1* (contains control Y14-knockdown nonresponsive intron) (Supplementary Figure S3C). These results strongly suggest that RNPS1 interacts with the EJC core and promotes the efficient and faithful pre-mRNA splicing of the target Y14-knockdown responsive introns.

## 3. Discussion

It has been recently shown that knockdown of the EJC core factor causes global alternative splicing changes in mammalian cells [21]. Our Y14-knockdown experiments followed by RNA-Seq analysis uncover an important new aspect of the EJC core function. We found that the EJC core contributes, not only to the efficiency, but also to the fidelity of constitutive splicing in a set of functionally related genes.

### 3.1. The EJC as a Master Splicing Controller of Genes Involved in Cell Cycle Progression

The EJC was documented to have multiple roles in the post-splicing events of mammalian gene expression (reviewed in [1]). Here, we demonstrate that the EJC core component Y14 is required for the efficient splicing of target introns in many genes involved in mitotic cell cycle progression. The Y14-knockdown derived intron-retention events were also accompanied by a variety of aberrant splicing, such as alternative splice site usage and exon skipping. The generation of these abnormal transcripts naturally leads to a reduction of the full-length transcripts. The abnormal transcripts may often be dead-end mRNAs that are destined to be degraded or they may even produce antagonistic or dominant negative proteins, which disturb correct mitotic cell cycle progression. Consistently, it was previously shown that depletion of Y14 results in G2/M cell cycle arrest followed by apoptosis [22]. Here, we observed that Y14 knockdown in HeLa cells induces abnormal nuclear structure, multinucleated cells, G2/M cell cycle arrest, and genome instability.

We identified various abnormal transcripts of *AURKB* (Aurora B kinase) and *MDM2* (murine double minute 2) genes in Y14-knockdown HeLa cells. AURKB, a serine/threonine kinase, functions in chromosome segregation, cleavage of polar spindle microtubules and cytokinesis [23]. Inhibition of the AURKB function in mitotic cells causes misaligned chromosomes and defective cytokinesis, which results in polyploidy (≥4N cells) [23,24]. MDM2 possesses E3 ubiquitin ligase activity that targets P53, and *MDM2* knockout in mouse germ line causes embryonic lethality at the blastocyst stage due to inappropriate apoptosis [25,26,27]. Thus, the known functions of MDM2 and AURKB would be able to explain a part of the phenotype caused by Y14 knockdown.

The changes in transcript level, alternative splicing, and protein level in the core EJC-deficient cells were reported to cause the disruption of proper cell cycle progression and the apoptosis process, which is indeed consistent with our results. Mouse *Magoh* mutant haplo-insufficiency causes the defect of mitosis of neural stem cells and apoptosis, which are rescued by restoring the expression of Lis1, a microtubule-associated protein essential for mitotic spindle integrity [18]. Moreover, a recent study demonstrated that the EJC components regulate the alternative splicing of several apoptotic genes; i.e., knockdown of the EJC core (Y14 and eIF4AIII) or splicing-related EJC peripheral (RNPS1, ACINUS and SAP18) proteins increases the production of the pro-apoptotic splice variant of *Bcl-x_S_* pre-mRNA [15]. Taking our findings and these studies together, we conclude that the EJC plays an important role in the expression of genes involved in proper cell cycle progression and apoptosis.

### 3.2. Molecular Mechanisms of EJC-Mediated Splicing Regulation

We found that the EJC core factor, Y14, controls splicing of a specific group of short-intron. Using the model PSMB4 pre-mRNA harboring intron 5 (186 nt), we showed that the EJC deposition near the intron is critical to stimulating splicing activity. Recent studies in *Drosophila* also indicated that efficient splicing of the *piwi* pre-mRNA containing intron 4 is promoted by RnpS1 and Acinus, which are recruited by the pre-deposited EJC at adjacent spliced exon junctions [28,29].

It was proposed that pre-mRNA splicing of short introns occurs by the formation of a splicing complex across the introns (termed “intron definition”), whereas that of long intron, a cross-exon splicing complex is formed (termed “exon definition”) prior to the splicing of adjacent introns [30,31]. Therefore, the deposit of the EJC may underpin precise short intron recognition and formation of a stable intron definition complex. Previously, it was shown that the EJC associates with several splicing regulators such as RNPS1, PININ, ACINUS, and SAP18, which contain specific domains that are capable of interacting with general splicing factors (reviewed in [1]). Here, we demonstrate that the EJC-peripheral factor, RNPS1 at least, is the *trans*-acting factor for the efficient splicing of pre-mRNAs containing Y14-knockdown responsive short introns. On the other hand, RNPS1 was reported to be associated with SAP18 and ACINUS, to form the apoptosis and splicing-associated protein (ASAP) complex [8]. Moreover, a recent structural analysis revealed that RNPS1 and SAP18 are able to interact with PININ, forming another ternary complex, PSAP [14]. Therefore, it remained to be elucidated whether RNPS1 solely or RNPS1 complex, such as ASAP (PSAP), is responsible for the EJC core mediated splicing activation.

**Figure 6:**
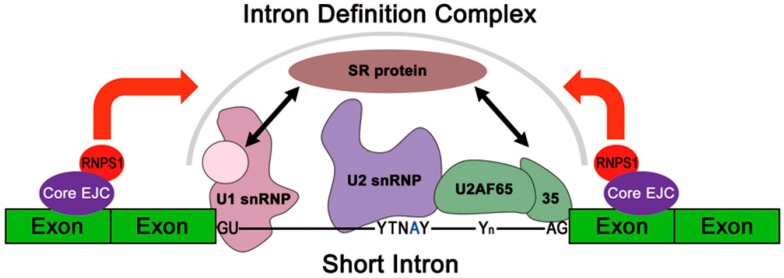
A model of EJC mediated splicing activation. RNPS1 and other EJC associated splicing regulators interact with the core EJC and recruit general splicing factors to essential splicing elements of specific short introns, leading to the efficient or stable formation of an intron definition complex.

Taken together, we propose the model that the initial deposition of the EJC core at adjacent (upstream and/or downstream) spliced junctions recruits EJC-peripheral splicing regulator(s), either RNPS1 alone or in the ASAP (PSAP) complex, to promote the efficient or stable formation of intron definition complexes on the proximate short intron (Figure 6). This finding provides a mechanistic link between the originally identified RNPS1 as a general splicing activator/regulator [2,3] and the detection of RNPS1 as a peripheral component of the EJC (reviewed in [1]).

Interestingly, we observed pre-mRNA splicing activation, not inhibition, by the Y14-knockdown experiment in another specific subset of pre-mRNAs, suggesting EJC-mediated repression rather than activation of splicing. In this opposite case, it will be interesting to ascertain what is the *trans*-acting factor recruited by EJC core to promote splicing repression. We wish to propose that the EJC core as potential master splicing controller with potential to recruit splicing regulators, either positive or negative, to define distinct splicing activity modes.

## 4. Experimental Procedures

### 4.1. Plasmid Constructions and Antibodies

To construct pCS2-HA-λN-eIF4AIII, pCS2-HA-λN-Y14, and pCS2 -HA-λN-MAGOH, cDNAs were amplified by PCR and subcloned into pCS2-HA-λN vector as previously described [32]. Five contiguous copies of the BoxB sequence were amplified by PCR from pCS2+Rluc-BoxB [32], and cloned into pcDNA3 (Thermo Fisher Scientific, Waltham, MA, USA). The pcDNA3-PSMB4-5BoxB mini-gene was obtained by subcloning PCR-amplified HeLa genomic DNA containing the exon 5, intron 5 and exon 6 region into the pcDNA3-5BoxB vector. The following antibodies were commercially available: anti-Y14 (Sigma, St. Louis, MO, USA), anti-UPF1 and anti α-tubulin (Cell Signaling Technology, Danvers, MA, USA), and anti-MDM2 and anti-AURKB (Abcam, Cambridge, UK). The anti-RNPS1 antibody was previously described [2].

### 4.2. Cell Culture and siRNA Knockdown

HeLa cells were maintained in Dulbecco’s modified Eagle’s medium supplemented with 10% fetal bovine serum. ON-TARGET plus SMART pool siRNA reagents and negative control siRNA (GE Healthcare, Chicago, IL, USA) were used to knockdown the expression of Y14, RNPS1 and UPF1. Transfection of the siRNA was performed with Lipofectamine RNAiMax (Thermo Fisher Scientific) according to the manufacture’s protocol. HeLa cells were grown in 35 mm dishes and transfected with each siRNA (100 pmol). At 48 h post-transfection, total proteins and RNAs were isolated from the siRNA-treated HeLa cells using ISOGEN (Wako, Kyoto, Japan).

### 4.3. RNA-Seq Analysis

For RNA-Seq, mRNA isolation and DNA library preparation were performed according to the manufacturer’s protocol (Illumina, San Diego, CA, USA). The DNA libraries were prepared from four independent RNA sources; HeLa cells treated with two control siRNAs and two Y14 siRNAs. These samples were sequenced on a high-throughput platform (HiSeq2000, Illumina) using a 76 bp single-end strategy. The reads were mapped onto the hg19 human genome sequences using the TopHat 1.12.0 (https://ccb.jhu.edu/software/tophat/index.shtml). All positions of junctions contained in the mapping results were annotated as an intron in the ENSEMBL annotation database (http://asia.ensembl.org/index.html). Our RNA-Seq analysis is shown in Supplementary Experimental Procedures. RNA-Seq raw data have been deposited in DDBJ database (http://www.ddbj.nig.ac.jp/index-e.html) under accession No. DRA004068.

### 4.4. Tethering Experiments

For the tethering assays, 0.1 µg of pcDNA3-PSMB4-5BoxB with 0.5 µg of pCS2-HA-λN-eIF4AIII, pCS2-HA-λN-Y14 or pCS2-HA-λN-MAGOH were co-transfected into UPF1 siRNA- or Y14 siRNA-treated HeLa cells in 35 mm dishes, using PolyFect transfection reagent (QIAGEN, Venlo, The Netherlands). Transfected HeLa cells were cultured for 24 h before extraction of proteins and RNAs. Expression level of HA-λN fusion proteins and endogenous UPF1 or Y14 protein were examined by Western blotting. To analyze splicing products from the *PSMB4* mini-gene, total RNA from transfected cells were analyzed by RT–PCR using T7 and PSMB4-E6AS-XhoI primers (Supplementary Table S4). PCR products were analyzed by 1.5% or 2% agarose gel electrophoresis. Splicing products were quantified using NIH Image J software (https://imagej.nih.gov/ij/) [33,34].

### 4.5. Immunoprecipitation Experiments

For immunoprecipitation of Y14 and eIF4E-associated mRNA, whole HeLa cell extracts were prepared and mixed with antibodies conjugated with Dynal beads protein G (Invitrogen, Carlsbad, CA, USA) in NET2 buffer [35]. After a 3 h incubation at 4 °C, the beads were washed six times with NET2 buffer and bound RNA was recovered by phenol extraction and ethanol precipitation. The precipitated RNAs were analyzed by RT–PCR.

## Supplementary Materials

Supplementary materials can be found at http://www.mdpi.com/1422-0067/17/8/1153/s1.

## References

[1] Le Hir H., Sauliere J., Wang Z. (2016). The exon junction complex as a node of post-transcriptional networks. Nat. Rev. Mol. Cell Biol..

[2] Mayeda A., Badolato J., Kobayashi R., Zhang M.Q., Gardiner E.M., Krainer A.R. (1999). Purification and characterization of human RNPS1: A general activator of pre-mRNA splicing. EMBO J..

[3] Sakashita E., Tatsumi S., Werner D., Endo H., Mayeda A. (2004). Human RNPS1 and its associated factors: A versatile alternative pre-mRNA splicing regulator in vivo. Mol. Cell. Biol..

[4] Trembley J.H., Tatsumi S., Sakashita E., Loyer P., Slaughter C.A., Suzuki H., Endo H., Kidd V.J., Mayeda A. (2005). Activation of pre-mRNA splicing by human RNPS1 is regulated by CK2 phosphorylation. Mol. Cell. Biol..

[5] Lykke-Andersen J., Shu M.D., Steitz J.A. (2001). Communication of the position of exon–exon junctions to the mRNA surveillance machinery by the protein RNPS1. Science.

[6] Viegas M.H., Gehring N.H., Breit S., Hentze M.W., Kulozik A.E. (2007). The abundance of RNPS1, a protein component of the exon junction complex, can determine the variability in efficiency of the Nonsense Mediated Decay pathway. Nucleic Acids Res..

[7] McCracken S., Longman D., Johnstone I.L., Caceres J.F., Blencowe B.J. (2003). An evolutionarily conserved role for SRm160 in 3’-end processing that functions independently of exon junction complex formation. J. Biol. Chem..

[8] Schwerk C., Prasad J., Degenhardt K., Erdjument-Bromage H., White E., Tempst P., Kidd V.J., Manley J.L., Lahti J.M., Reinberg D. (2003). ASAP, a novel protein complex involved in RNA processing and apoptosis. Mol. Cell. Biol..

[9] Joselin A.P., Schulze-Osthoff K., Schwerk C. (2006). Loss of Acinus inhibits oligonucleosomal DNA fragmentation but not chromatin condensation during apoptosis. J. Biol. Chem..

[10] Zhang Y., Iratni R., Erdjument-Bromage H., Tempst P., Reinberg D. (1997). Histone deacetylases and SAP18, a novel polypeptide, are components of a human Sin3 complex. Cell.

[11] Singh K.K., Erkelenz S., Rattay S., Dehof A.K., Hildebrandt A., Schulze-Osthoff K., Schaal H., Schwerk C. (2010). Human SAP18 mediates assembly of a splicing regulatory multiprotein complex via its ubiquitin-like fold. RNA.

[12] Ouyang P., Sugrue S.P. (1996). Characterization of pinin, a novel protein associated with the desmosome-intermediate filament complex. J. Cell Biol..

[13] Wang P., Lou P.J., Leu S., Ouyang P. (2002). Modulation of alternative pre-mRNA splicing in vivo by pinin. Biochem. Biophys. Res. Commun..

[14] Murachelli A.G., Ebert J., Basquin C., Le Hir H., Conti E. (2012). The structure of the ASAP core complex reveals the existence of a Pinin-containing PSAP complex. Nat. Struct. Mol. Biol..

[15] Michelle L., Cloutier A., Toutant J., Shkreta L., Thibault P., Durand M., Garneau D., Gendron D., Lapointe E., Couture S. (2012). Proteins associated with the exon junction complex also control the alternative splicing of apoptotic regulators. Mol. Cell. Biol..

[16] Sauliere J., Haque N., Harms S., Barbosa I., Blanchette M., Le Hir H. (2010). The exon junction complex differentially marks spliced junctions. Nat. Struct. Mol. Biol..

[17] Roignant J.Y., Treisman J.E. (2010). Exon junction complex subunits are required to splice *Drosophila* MAP kinase, a large heterochromatic gene. Cell.

[18] Silver D.L., Watkins-Chow D.E., Schreck K.C., Pierfelice T.J., Larson D.M., Burnetti A.J., Liaw H.J., Myung K., Walsh C.A., Gaiano N. (2010). The exon junction complex component Magoh controls brain size by regulating neural stem cell division. Nat. Neurosci..

[19] Nothwang H.G., Tamura T., Tanaka K., Ichihara A. (1994). Sequence analyses and inter-species comparisons of three novel human proteasomal subunits, HsN3, HsC7-I and HsC10-II, confine potential proteolytic active-site residues. Biochim. Biophys. Acta.

[20] Gehring N.H., Kunz J.B., Neu-Yilik G., Breit S., Viegas M.H., Hentze M.W., Kulozik A.E. (2005). Exon-junction complex components specify distinct routes of nonsense-mediated mRNA decay with differential cofactor requirements. Mol. Cell.

[21] Wang Z., Murigneux V., Le Hir H. (2014). Transcriptome-wide modulation of splicing by the exon junction complex. Genome Biol..

[22] Ishigaki Y., Nakamura Y., Tatsuno T., Hashimoto M., Shimasaki T., Iwabuchi K., Tomosugi N. (2013). Depletion of RNA-binding protein RBM8A (Y14) causes cell cycle deficiency and apoptosis in human cells. Exp. Biol. Med. (Maywood).

[23] Nair J.S., Ho A.L., Tse A.N., Coward J., Cheema H., Ambrosini G., Keen N., Schwartz G.K. (2009). Aurora B kinase regulates the postmitotic endoreduplication checkpoint via phosphorylation of the retinoblastoma protein at serine 780. Mol. Biol. Cell.

[24] Nigg E.A. (2001). Mitotic kinases as regulators of cell division and its checkpoints. Nat. Rev. Mol. Cell Biol..

[25] Manfredi J.J. (2010). The Mdm2-p53 relationship evolves: Mdm2 swings both ways as an oncogene and a tumor suppressor. Genes Dev..

[26] Jones S.N., Roe A.E., Donehower L.A., Bradley A. (1995). Rescue of embryonic lethality in Mdm2-deficient mice by absence of p53. Nature.

[27] Montes de Oca Luna R., Wagner D.S., Lozano G. (1995). Rescue of early embryonic lethality in *mdm2*-deficient mice by deletion of *p53*. Nature.

[28] Hayashi R., Handler D., Ish-Horowicz D., Brennecke J. (2014). The exon junction complex is required for definition and excision of neighboring introns in *Drosophila*. Genes Dev..

[29] Malone C.D., Mestdagh C., Akhtar J., Kreim N., Deinhard P., Sachidanandam R., Treisman J., Roignant J.Y. (2014). The exon junction complex controls transposable element activity by ensuring faithful splicing of the piwi transcript. Genes Dev..

[30] Berget S.M. (1995). Exon recognition in vertebrate splicing. J. Biol. Chem..

[31] Wahl M.C., Will C.L., Luhrmann R. (2009). The spliceosome: Design principles of a dynamic RNP machine. Cell.

[32] Mishima Y., Fukao A., Kishimoto T., Sakamoto H., Fujiwara T., Inoue K. (2012). Translational inhibition by deadenylation-independent mechanisms is central to microRNA-mediated silencing in zebrafish. Proc. Natl. Acad. Sci. USA.

[33] Fukumura K., Taniguchi I., Sakamoto H., Ohno M., Inoue K. (2009). U1-independent pre-mRNA splicing contributes to the regulation of alternative splicing. Nucleic Acids Res..

[34] Fukumura K., Kato A., Jin Y., Ideue T., Hirose T., Kataoka N., Fujiwara T., Sakamoto H., Inoue K. (2007). Tissue-specific splicing regulator Fox-1 induces exon skipping by interfering E complex formation on the downstream intron of human F1γ gene. Nucleic Acids Res..

[35] Ideue T., Sasaki Y.T., Hagiwara M., Hirose T. (2007). Introns play an essential role in splicing-dependent formation of the exon junction complex. Genes Dev..

[36] Shibuya T., Tange T.O., Sonenberg N., Moore M.J. (2004). eIF4AIII binds spliced mRNA in the exon junction complex and is essential for nonsense—Mediated decay. Nat. Struct. Mol. Biol..

[37] Trapnell C., Williams B.A., Pertea G., Mortazavi A., Kwan G., van Baren M.J., Salzberg S.L., Wold B.J., Pachter L. (2010). Transcript assembly and quantification by RNA-Seq reveals unannotated transcripts and isoform switching during cell differentiation. Nat. Biotechnol..

[38] Roberts A., Trapnell C., Donaghey J., Rinn J.L., Pachter L. (2011). Improving RNA-Seq expression estimates by correcting for fragment bias. Genome Biol..

[39] Roberts A., Pimentel H., Trapnell C., Pachter L. (2011). Identification of novel transcripts in annotated genomes using RNA-Seq. Bioinformatics.

