# Supplementary material for: The Exon Junction Complex Controls the Efficient and Faithful Splicing of a Subset of Transcripts Involved in Mitotic Cell-Cycle Progression
Source: Int J Mol Sci. 2016 Aug 2;17(8):1153. doi: 10.3390/ijms17081153 (PMC5000587; doi:10.3390/ijms17081153)
Supplement: Supplementary file 1 [file ijms-17-01153-s001.zip › ijms-133856-Supplementary Materials/ijms-133856-Supplementary.pdf]

Supplementary Materials: The Exon Junction Complex Controls the Efficient and Faithful Splicing of a Subset of Transcripts Involved in Mitotic Cell-Cycle Progression

Kazuhiro Fukumura, Shunichi Wakabayashi, Naoyuki Kataoka, Hiroshi Sakamoto, Yutaka Suzuki, Kenta Nakai, Akila Mayeda and Kunio Inoue

Supplementary Experimental Procedures

Antibodies and siRNA

We used the following commercially available antibodies: anti-γH2A.X (Ser139) (Cell Signaling Technology, Danvers, MA, USA), ant-U2AF65 antibody (Sigma, St. Louis, MO, USA), and anti-SF3b155 (MBL, Nagoya, Japan). Anti-U1-70K antibody was a gift from S. I. Gunderson. The siRNA for eIF4A3 ([36], see References in the text) was synthesized by Sigma Genosys.

Immunostaining

HeLa cells were fixed with 3% formaldehyde in phosphate buffered saline (PBS), permeabilized with 0.1% Triton X–100 in PBS, blocked with BLOCK ACE (DS Pharma Biomedical, Osaka, Japan), and then incubated with primary antibodies in 2% bovine serum albumin in PBS for 0.5 h. After three successive washes with PBS, cells were incubated with Anti–Alexa Fluor 488 or Anti–Alexa Fluor 568 secondary antibodies (Invitrogen, Carlsbad, CA, USA), and then washed five times with PBS. The images were analyzed with a Fluorescence Microscope (Olympus, Tokyo, Japan).

FACS Analysis

HeLa cells were transfected with control siRNA and Y14 siRNA. After a 48 h culture, Hela cells were harvested and washed with PBS and suspended in 25 mg/mL propdium iodide (PI) in PBS with RNase A (200 μg/mL). Flow cytometry was performed with a FACScalibur system (BD Biosciences, Franklin Lakes, NJ, USA) followed by analysis using CellQuest Pro software (BD Biosciences).

Prediction of the Retained Intron from RNA-Seq Data Set

Intron Retention Rate (IRR) is the ratio of the read density of 20 nt regions in upstream/downstream exons (see figure in next page; shown as **A**) and the read density in the target intron (shown as **B**). To identify the retained intron in Y14 down- regulated HeLa cells, fold changes of IRR ($\log_2$ Y14 RNAi IRR/Control RNAi IRR) were calculated for the each intron. The retained introns in Y14 siRNA treated HeLa cells were defined as a fold change of IRR ratio >1. And the retained introns in control siRNA treated HeLa cells were defined as a fold change of IRR ratio < –1. A p-value control (<0.05) was applied for the fold change of IRR ratio.

Intron Length and Gene Ontology (GO) Analysis

Expression levels of all annotated genes were examined on the FPKM (i.e., fragments per kilobase of exon per million mapped sequence reads) scale by using Cufflinks 2.0.0 [37–39]. The gene set (>3.16 FPKM: 9801 genes) was defined as expressed genes in both of Y14 siRNA and control siRNA-treated HeLa cells. This expressed gene set includes the 99% of the retained introns containing genes in Y14 siRNA or control siRNA-treated HeLa cells. Intron length information in

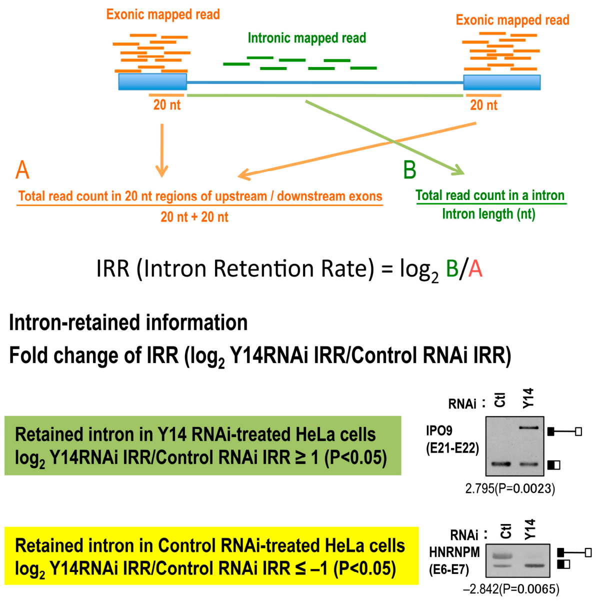

the RefSeq database was extracted from this expressed gene set. The GO terms of these expressed genes were examined by using the Ensembl database (http://www.ensembl.org/index.html). GO term enrichment analysis was preformed by comparing between this expressed genes and retained intron containing genes in Y14 knockdown HeLa cells. The 9636 GO terms were applied analysis using the Fisher exact test and the Bonferroni multivariate correction.

Supplementary Tables and Figures (see the separate 'Excel' file for Table S1A–D)

Table S2. Size distribution of the Y14 regulated introns.

Intron Length (nt)	Splicing Inhibition in Y14-KD (Ratio)	Splicing Activation in Y14-KD (Ratio)	Reference Intron (Ratio)	Splicing Inhibition in Y14-KD (Counts)	Splicing Activation in Y14-KD (Counts)	Reference Intron (Counts)
< 100	0.126	0.057	0.049	79	19	6787
< 200	0.158	0.143	0.076	99	48	10427
< 300	0.093	0.066	0.050	58	22	6915
< 400	0.080	0.063	0.040	50	21	5443
< 500	0.067	0.042	0.035	42	14	4850
< 600	0.046	0.048	0.032	29	16	4410
< 700	0.040	0.039	0.029	25	13	4039
< 800	0.026	0.048	0.028	16	16	3788
< 900	0.022	0.048	0.026	14	16	3521
< 1000	0.027	0.024	0.024	17	8	3339
< 1100	0.019	0.018	0.023	12	6	3211
< 1200	0.016	0.039	0.021	10	13	2916
< 1300	0.021	0.018	0.020	13	6	2759
< 1400	0.013	0.018	0.019	8	6	2669
< 1500	0.011	0.024	0.018	7	8	2451
< 1600	0.016	0.009	0.017	10	3	2346
< 1700	0.014	0.003	0.016	9	1	2222
< 1800	0.010	0.003	0.015	6	1	2055
< 1900	0.010	0.015	0.014	6	5	1946
< 2000	0.006	0.024	0.014	4	8	1928
< 3000	0.059	0.069	0.104	37	23	14308
< 4000	0.038	0.036	0.068	24	12	9328
< 5000	0.014	0.027	0.045	9	9	6185
≥ 5000	0.067	0.122	0.213	42	41	29273
**Total Numbers**				626	335	137116

Table S3. GO analysis of 483 genes that showed intron retention in Y14 knockdown HeLa cells.

ID	Count	Fold Change	*p*-Value	Gene Ontology (GO) Terms
GO:00000278	33	2.43	0.000020	Mitotic cell cycle
GO:00044267	31	2.51	0.000028	Cellular protein metabolic process
GO:00000075	17	2.80	0.000377	Cell cycle checkpoint
GO:00010467	35	2.00	0.000429	Gene expression
GO:00055086	11	3.66	0.000555	Nucleobase-containing small molecule metabolic process
GO:00051436	11	3.55	0.000702	Negative regulation of ubiquitin-protein ligase activity involved in mitotic cell cycle
GO:00006606	8	4.70	0.000757	Protein import into nucleus
GO:00006200	12	3.26	0.000797	ATP catabolic process
GO:00006611	6	6.48	0.000858	Protein export from nucleus
GO:00044419	26	2.11	0.000931	Interspecies interaction between organisms
GO:00031145	12	3.18	0.000976	Anaphase-promoting complex-dependent proteasomal ubiquitin-dependent protein catabolic process
GO:00045910	4	11.68	0.001218	Negative regulation of DNA recombination
GO:00007411	19	2.35	0.001364	Axon guidance
GO:00051439	11	3.15	0.001651	Regulation of ubiquitin-protein ligase activity involved in mitotic cell cycle
GO:00007568	11	3.02	0.002205	Aging
GO:00016032	27	2.00	0.002279	Viral reproduction
GO:00008340	4	9.09	0.002448	Determination of adult lifespan
GO:00016447	4	9.09	0.002448	Somatic recombination of immunoglobulin gene segments
GO:00050771	4	9.09	0.002448	Negative regulation of axonogenesis
GO:00000087	12	2.81	0.002451	M phase of mitotic cell cycle
GO:00000082	16	2.42	0.002801	G1/S transition of mitotic cell cycle
GO:00032727	3	15.30	0.003127	Positive regulation of interferon-α production
GO:00000236	11	2.87	0.003170	Mitotic prometaphase
GO:00043297	4	8.18	0.003301	Apical junction assembly
GO:00051437	10	3.03	0.003393	Positive regulation of ubiquitin-protein ligase activity involved in mitotic cell cycle
GO:00006302	9	3.19	0.003897	Double-strand break repair
GO:00000084	13	2.48	0.004388	S phase of mitotic cell cycle
GO:00009987	5	5.39	0.004529	Cellular process
GO:00000904	3	12.25	0.004830	Cell morphogenesis involved in differentiation
GO:00015886	3	12.25	0.004830	Heme transport

Table S4. The primers used in the experiments.

Primer names	Primer sequences (5′ to 3′)
IPO9-E21S	ACCCAGAACGCTGGACAAAC
IPO9-E22AS	GTTGGCCAGCTAAACCATCCT
NOP2-E15S	GAGAAGGACATCCTGCGCTG
NOP2-E16AS	CTGTCTGGGACTGAGGGATAG
NAE1-E12S	AGTCTTGTCTCACTGGCTTCC
NAE1-E13AS	GCAGCAATGGTATGTGGCTC
TP53BP1-E27S-EcoRI	CCGCTCGAGCCCAGTTACTACAACCAAAG
TP53BP1-E28AS-XhoI	CCGCTCGAGCCCAGTTACTACAACCAAAG
PC-E17S-EcoRI	GGAATTCTGCACCATGCTGGTCAGCTC
PC-E18AS-XhoI	CCGCTCGAGATGAGATCGCCCAGCATCTG
MAP4K4-E23S-EcoRI	GGAATTCATCCAGTGTAGCATCAAACC
MAP4K4-E24AS-XhoI	CCGCTCGAGTTGTCATTGCGTTCACACAA
CK2-E5S-EcoRI	GGAATTCACCAGCAAGGAGACTTTGGT
CK2-E6AS-XhoI	CCGCTCGAGACAAACTGGTTGGCAGGTCT
TUBG1-E8S-EcoRI	GGAATTCTGAGGAAGACCACGGTCCTG
TUBG1-E9AS-XhoI	CCGCTCGAGAGGAGATGCTGGTGTGGTTG
PSMC5-E9S-EcoRI	CGGGATCCGTTATCATGGCTACTAATAG
PSMC5-E10AS-XhoI	CCGCTCGAGCTTCACTTCAGCCCCTGATG
PSMD2-E17S-EcoRI	GGAATTCCTGAGATATGGGGAGCCTAC
PSMD2-E18AS-XhoI	CCGCTCGAGCTGTGCCAAGCGCACCATGA
CDK2-E6S-BamHI	CGGGATCCCCCTGGAGATTCTGAGATTG
CDK2-E7AS-XhoI	CCGCTCGAGCAAGACTAGAAGGTGAGTGT
CDK9-E5S-EcoRI	GGAATTCATCCTGCATAGGGACATGAA
CDK9-E6AS-XhoI	CCGCTCGAGATGGAGCCGCAGAGCTGACT
TALDO1-E6S	GGGTAAAGAGTGTCACTAA
TALDO1-E7AS	TGTCAGCATCCGCTCCAGCT
TMEM147-E4S	GAGTTCATGAAGGCCAGCGT
TMEM147-E5AS	CAGACTGATGTTGGAGTCT
AURKB-E5S-EcoRI	GGAATTCGGCACTTCACAATTGATGAC
AURKB-E6AS-XhoI	CCGCTCGAGGTCAAATGTGCAGCTCTTCT
NUP85-E17S-EcoRI	GGAATTCGAAAGTATCGCGAGTTCCAC
NUP85-E18AS-XhoI	CCGCTCGAGGCTGCTCGGTATCAGATTCT
PSMB4-E5S-EcoRI	GGAATTCCCTCTGCTGCGAGAAGTTCT
PSMB4-E6AS-XhoI	CCGCTCGAGCTGATCATGTGGGCAATATC
POLR2C-E7S-EcoRI	GGAATTCACATCCTCATCGTCAAGTTG
POLR2C-E8AS-XhoI	CCGCTCGAGCTTTCTGGCTTGCCGTTGGG
MDM2E9S-EcoRI	GGAATTCCGCTGGTGTAAGTGAACATTC
MDM2E10AS-XhoI	CCGCTCGAGGCTAAGGAAATTTCAGGATC
ACADVLE9S-BamHI	CGGGATCCCTAGCAGACATCTTCACGGT
ACADVLE10AS-NotI	GAGCGGCCGCAATGATGCCTCTCATGGTAC
hnRNPM-E5S-BamHI	CGGGATCCTGTTGTTGAATTCAAGATGG
hnRNPM-E8AS-XhoI	CCGCTCGAGATATAGCTTGCACAGCTTCA
CDK1-E5S-EcoRI	GGAATTCGTAGTAACACTCTGGTACAG
CDK1-E6AS-XhoI	CCGCTCGAGCGAGAGCAAATCCAAGCCAT
ACTG1-E3-S	TGACCCTGAAGTACCCCATT
ACTG1-E4-AS	TGGTGGTGAAGCTGTAGCCT
MDM2-E3-S-EcoRI	CGGAATTCCGCGAAAACCCCGGGCAGGCAAATGTGCA
MDM2-E12-AS-XbaI	CTAGTCTAGACTCTTATAGACAGGTCAACTAG
AURKB-E2-S	TAAGGATGGCCCAGAAGGAG
AURKB-E9-AS	GGAACAGTTAGGGATCCCTT
ACTG1-E2S	GCTGGTCATTGACAATGGCT
ACTG1-E6AS	ATTTGCGGTGGACGATGGAG
Y14-S-XhoI	CCGCTCGAGATGGCGGACGTGCTAGATCT
Y14-AS-XbaI	GCTCTAGATCACCTCTTGCCTTTTGGTG-3′
MAGOH-S-XhoI	CCGCTCGAGATGGAGAGTGACTTTTATCT
MAGOH-AS-XbaI	GCTCTAGACTAGATTGGTTTAATCTTGA
eIF4A3-S-XhoI	CCGCTCGAGATGGCGACCACGGCCACGAT
eIF4A3-AS-XbaI	GCTCTAGATCAGATAAGATCAGCAACGT
GAPDH-E2S	ACCATGGGGAAGGTGAAGGT
GAPDH-E9AS	TCCACCACCCTGTTGCTGTA

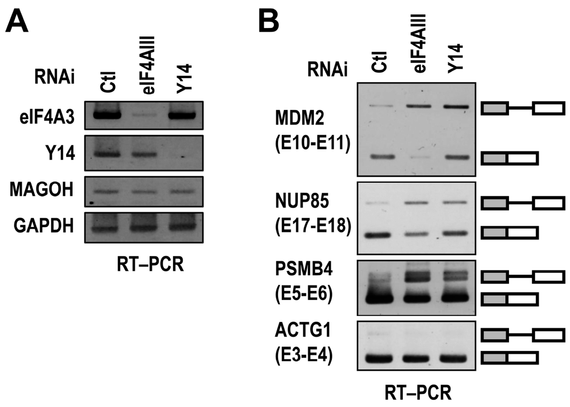

Figure S1. The eIF4A3 is required for splicing of pre-mRNA with Y14-regulated introns. HeLa cells were transfected with control siRNA or Y14 siRNA. At 48 h post-transfection, total RNAs were extracted and analyzed. (**A**) Knockdown efficiencies of Y14 or eIF4AIII were checked by RT–PCR; (**B**) Splicing efficiencies of pre-mRNA with Y14-regulated introns were analyzed by RT–PCR using specific primer sets (shown in Table S4).

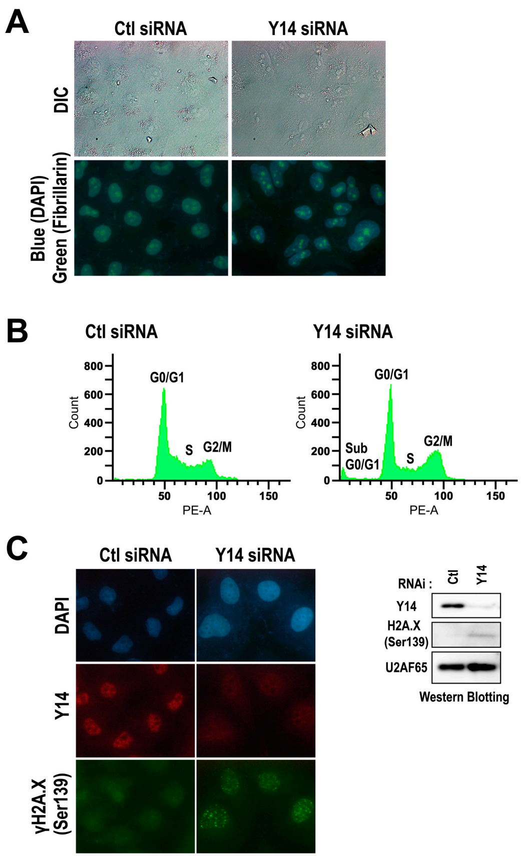

Figure S2. Y14 knockdown causes abnormal nuclear structures, G2/M arrest, and DNA double strand breaks. (**A**) HeLa cells were transfected with control (Ctl) siRNA or Y14 siRNA. At 48 h post-transfection, cells were immunostained with fibrillarin antibody together with DAPI; (**B**) HeLa cells were transfected with control (Ctl) siRNA or Y14 siRNA. At 48 h post-transfection, DNA content was measured by propidium iodide staining and flow cytometric analysis; (**C**) Control (Ctl) siRNA- or Y14 siRNA-treated HeLa cells were stained with Y14 antibody or γH2A.X (Ser139) antibody (left panel). Whole cell extracts of control siRNA- or Y14 siRNA-treated HeLa cells were analyzed by Western blotting using the same antibodies (right panel).

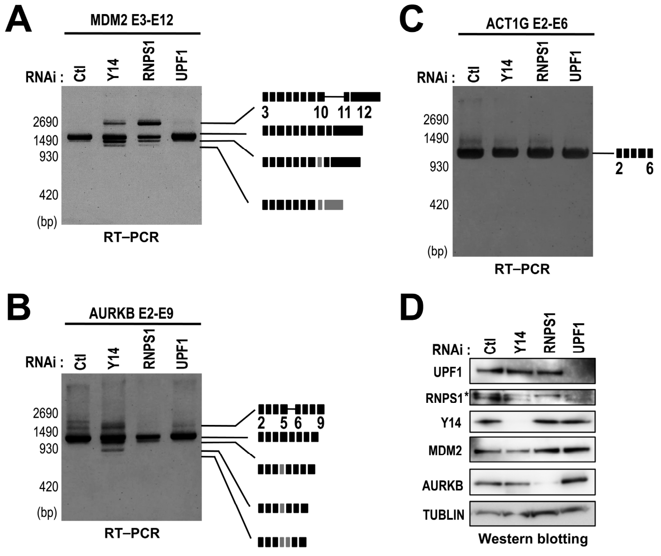

Figure S3. RNPS1 is required for faithful splicing of *MDM2* and *AURKB* mRNA. (**A**–**C**) HeLa cells were transfected with control siRNA, Y14 siRNA, RNPS1 siRNA, or UPF1 siRNA. At 48 h post-transfection, total RNAs were isolated and analyzed by RT–PCR using specific primer sets for MDM2, AURKB and ACTG1 (shown in Table S4). RT–PCR products were subcloned and the sequences were verified. The schematic representation at the right of each panel indicates the corresponding mRNA structures. Black boxes represent full-length exons and grey boxes represent truncated exons generated by alternative splice site usage; (**D**) Western blot analysis of whole cell extract from siRNAs-treated HeLa cells using anti-UPF1, anti-RNPS1, anti-Y14, anti-MDM2, anti-AURKB, and anti-TUBLIN antibodies. Asterisk (*) indicates the non-specific signal.
